# Hepatogastric Fistula as a Rare Complication of Pyogenic Liver Abscess: A Case Report

**DOI:** 10.1002/ccr3.71331

**Published:** 2025-10-20

**Authors:** Mohammad Kazem Amirbeigy, Amir Pasha Amel Shahbaz, Zahra Sekandari, Shahab Sheikhalishahi

**Affiliations:** ^1^ Department of Gastroenterology Shahid Sadoughi University of Medical Sciences Yazd Iran; ^2^ Department of Radiology Shahid Sadoughi University of Medical Sciences Yazd Iran; ^3^ Cardiology Resident Shahid Sadoughi University of Medical Sciences Yazd Iran; ^4^ Student Research Committee Shahid Sadoughi University of Medical Sciences Yazd Iran

**Keywords:** case report, cholangitis, hepatogastric fistula (HGF), pyogenic liver abscess (PLA)

## Abstract

Hepatogastric fistula is a rare complication of pyogenic liver abscess. We report an 18‐year‐old male with 3 weeks of fever, melena, and epigastric discomfort. Ultrasound showed a contracted gallbladder with wall thickening and sludge without cholelithiasis. Triphasic CT demonstrated a solid–cystic collection in hepatic segments IV and V abutting the gastric antrum, compatible with a fine communication. Esophagogastroduodenoscopy revealed a 3 mm antral mucosal orifice with purulent drainage. Smear showed neutrophil‐predominant exudate and Gram‐positive cocci; culture yielded *Staphylococcus aureus* susceptible to ciprofloxacin and clindamycin. Therapy was narrowed to ciprofloxacin 400 mg twice daily and clindamycin 600 mg three times daily. A 14‐day *Helicobacter pylori* eradication regimen was completed. Subsequently, laparoscopic cholecystectomy was performed. Intraoperatively the gallbladder was contracted with an inflamed but intact wall containing sludge; Calot's triangle was edematous yet the critical view of safety was obtained, and no cholecystoenteric fistula was identified. No drainable hepatic collection was present, consistent with decompression through the antral orifice. Opening the specimen demonstrated microlithiasis. Recovery was uneventful, and the patient remained with normal laboratory indices at 3 months. This case highlights the complementary roles of cross‐sectional imaging and endoscopy and supports individualized management in young patients with biliary‐source liver abscess.

**Abbreviations**
CT: computed tomography
EGD: esophagogastroduodenoscopy
HGF: hepatogastric fistula
PCD: percutaneous catheter drainage
PLA: pyogenic liver abscess
RBC: red blood cells
WBC: white blood cells

**Summary**

- Hepatogastric fistula is an exceptionally rare complication of pyogenic liver abscess. In young patients with atypical symptoms, CT may be suggestive while endoscopy confirms gastric involvement.
- Multidisciplinary care—image‐guided drainage, elective cholecystectomy, and culture‐directed therapy—can be curative, achieving sustained recovery even when a discrete fistulous tract is not depicted on CT.

## 1 Introduction

Pyogenic liver abscess (PLA) is an uncommon but potentially life‐threatening infection that most often arises from biliary tract disease, portal spread, or hematogenous seeding [1]. Despite advancements in imaging and antibiotic therapy, complications may still occur, particularly in delayed or untreated cases [2]. Rupture into adjacent spaces is recognized; however, fistulization into the gastrointestinal tract, particularly a hepatogastric fistula (HGF), is exceptionally rare and described mainly in single‐case reports [3]. Disease‐specific guidelines for HGF are lacking, and management is therefore individualized according to hemodynamic stability, abscess characteristics, and concurrent biliary pathology [4]. We report HGF in an 18‐year‐old male—an age group in which PLA is unusual—where endoscopy demonstrated a small antral orifice with purulent drainage, while cross‐sectional imaging showed no discrete tract. We outline the diagnostic reasoning using the Tokyo Guidelines framework, discuss plausible etiologies, and describe successful management with image‐guided drainage, elective cholecystectomy for source control, and culture‐directed antistaphylococcal therapy.

## 2 Case History/Examination

An 18‐year‐old male presented to our center with a 3‐week history of intermittent high‐grade fever, chills, nausea, and vomiting. Approximately 1 week after the onset of symptoms, he developed melena, prompting him to seek medical attention in his hometown, where he was hospitalized for 16 days. During this hospitalization, his hemoglobin level was recorded at 5.5 g/dL, and he received three units of packed red blood cells as part of supportive management. He was discharged at his own request after partial improvement, but due to persistent fatigue and incomplete recovery, he sought further evaluation at our facility. On admission, he reported ongoing melena, nausea, vomiting, mild upper abdominal discomfort, poor appetite, and generalized weakness. He denied any changes in bowel habits and has no history of past medical conditions. In terms of social history, the patient comes from a low socioeconomic background and has limited educational attainment. His medical and family history is unremarkable. He has no known history of chronic illnesses or significant prior medical conditions. Additionally, there is no significant history of changes in bowel function or any other alarming symptoms.

On physical examination, the patient was hemodynamically stable with stable vital signs. His general appearance was notable for pallor and mild malaise. Abdominal examination revealed mild tenderness localized to the epigastric region, without guarding, rigidity, or signs of peritonitis. There was no rebound tenderness, and bowel sounds were present and normoactive. No hepatosplenomegaly or palpable masses were detected. The remainder of the systemic examination was unremarkable.

## 3 Investigations and Treatment

Upon admission, initial laboratory investigations were promptly ordered, the results of which are summarized in Table 1. A complete abdominal and pelvic ultrasound was performed. The ultrasound revealed an enlarged liver measuring 165 mm with normal parenchymal echogenicity. Multiple heteroechoic structures were noted in the left lobe of the liver, some of which appeared confluent and exerted mass effect on the adjacent gallbladder. The largest of these lesions measured approximately 30 × 23 mm and raised suspicion for a possible neoplastic process; however, an infectious etiology could not be definitively excluded at this stage. The gallbladder was contracted, with diffuse wall thickening up to 7 mm and associated peripheral fat stranding, findings that may suggest an underlying inflammatory process. Additionally, compacted echogenic sludge measuring approximately 5 × 15 mm was observed within the gallbladder lumen. No gallstones were identified. The proximal portion of the common bile duct demonstrated a normal diameter.

**TABLE 1:** Laboratory tests.

CBC		LFT	
WBC	5500/cumm	AST	121 IU/L
RBC	3.68 Mil/cumm	ALT	125 IU/L
Hemoglobin	10.5 g/dL	Total bilirubin	0.3 mg/dL
Hematocrit	32.8%	Direct bilirubin	0.1 mg/dL
MCV	89.13 fL	ALP	560 IU/L
MCH	26.1 pg	Inflammatory factors	
MCHC	29.32 g/dL	CRP	1+
Platelets	570,000/cumm	ESR_1 hr	111 mm/h
Ferritin	896.3 ng/mL		

Abbreviations: ALP, alkaline phosphatase; ALT, alanine aminotransferase; AST, aspartate aminotransferase; CBC, complete blood count; CRP, C‐reactive protein; ESR, erythrocyte sedimentation rate; MCH, mean corpuscular hemoglobin; MCHC, mean corpuscular hemoglobin concentration; MCV, mean corpuscular volume; RBC, red blood cells; WBC, white blood cells.

**FIGURE 1:**
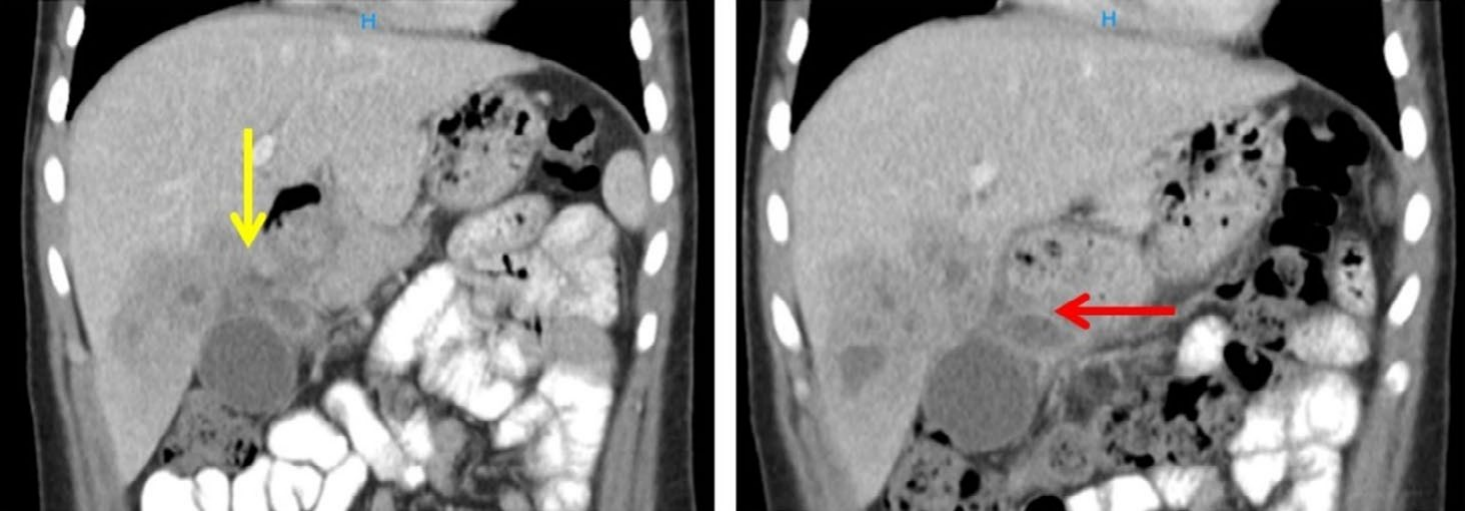
Coronal CT shows a multiloculated hepatic abscess adjacent to the gastric antrum (yellow arrow). While a fistulous communication is suspected based on topology and endoscopic correlation, a discrete tract is not visualized on CT. A dependent perigastric fluid collection is seen inferior to the antrum (red arrow).

**FIGURE 2:**
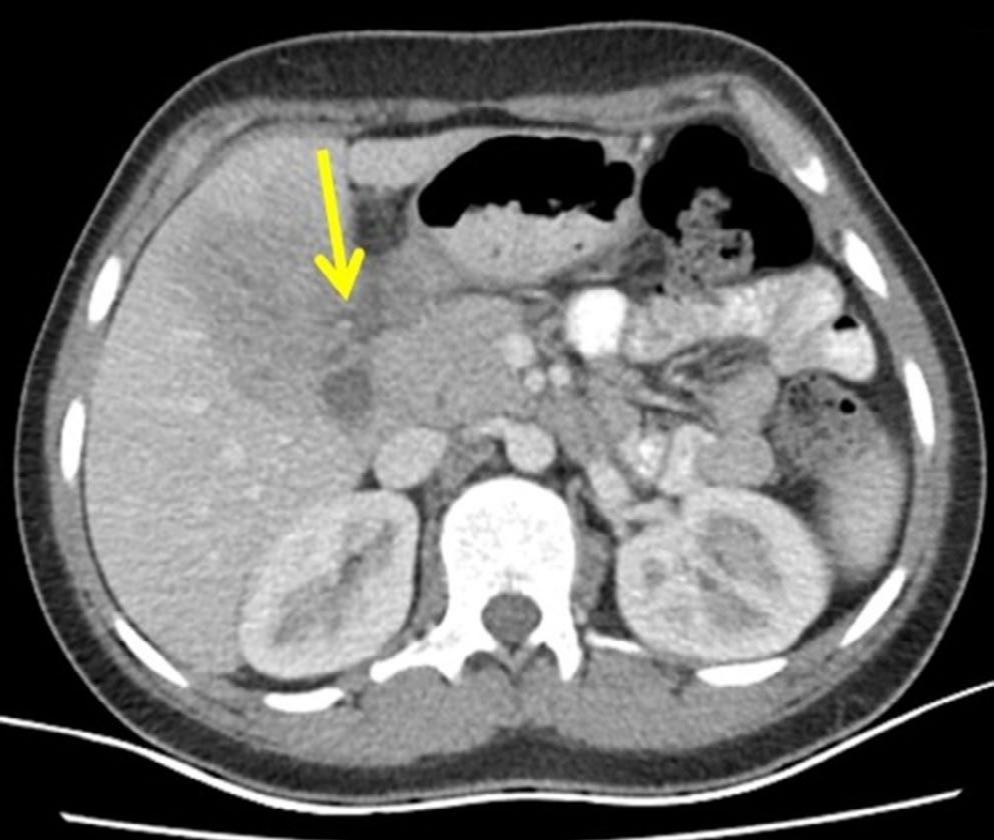
Axial CT demonstrates a multiloculated abscess in the left hepatic lobe (yellow arrow) abutting the gastric antrum, raising suspicion for fistulization.

A recommendation was made for further evaluation with a triphasic CT scan, which demonstrated a solid—cystic enhancing lesion in hepatic segments IV and V measuring 84 × 74 × 58 mm with loss of perihepatic and periantral fat planes, in keeping with abscess formation. A peripheral enhancing collection measuring 26 × 16 mm was noted inferior to the gastric antrum, with mass effect around the gallbladder. No cholecystogastric tract or features of gallbladder perforation were identified on CT. The hepatic collection closely abutted the anterior gastric wall at the antrum with minimal intervening fat; no discrete fistulous tract was visualized on CT, but the appearance was suggestive of a very fine communication (Figures 1 and 2).

To further investigate the underlying etiology of the hepatic lesion and the accompanying systemic inflammatory response, an extended panel of laboratory tests was conducted on the fourth day of hospitalization. This comprehensive workup was aimed at evaluating possible infectious, hematologic, and immunologic causes. It included serologic testing for viral hepatitis and HIV (HBsAg, anti‐HCV, and HIV antibody), as well as markers of hemolysis such as lactate dehydrogenase, reticulocyte count, and the direct Coombs test to assess for hemolysis. All viral serologies returned negative. The reticulocyte count was elevated (6.2%), and the hemoglobin level was decreased (9.6 g/dL), both findings consistent with hemolysis.

**FIGURE 3:**
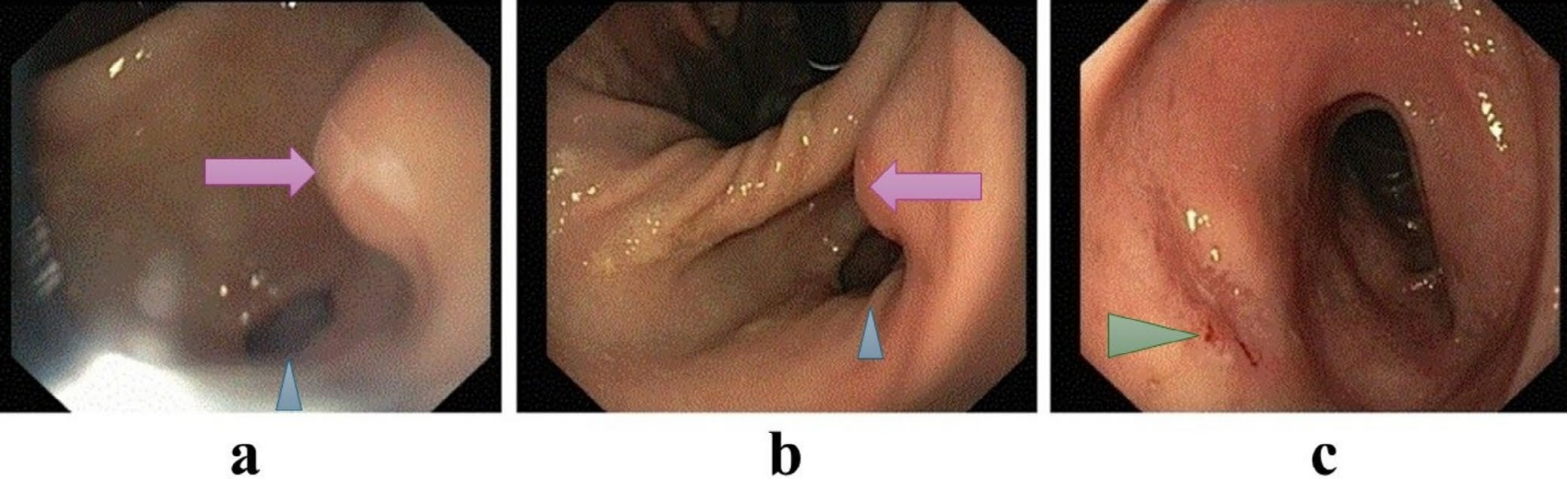
Gastroscopy revealed a fistulous opening in the antrum of the stomach (purple arrow in a and b), with the pylorus identified by the blue arrowhead. The incisura angularis is visible centrally in image (b). In image (c), a duodenal ulcer is seen on the anterior wall of the bulb (green arrowhead).

Esophagogastroduodenoscopy (EGD) was performed to evaluate a potential gastrointestinal source of bleeding, infection, or fistulous communication in the setting of melena, unexplained anemia, and persistent systemic inflammation. EGD identified a 3–4 mm antral mucosal orifice with active purulent drainage, consistent with a fistulous opening; the upstream origin of the tract could not be determined endoscopically (Figure 3a,b). The examination also identified a duodenal ulcer without active bleeding (Figure 3c). A sample from the fistulous orifice was obtained for microbiological smear and culture. Direct evaluation demonstrated a neutrophil‐predominant suppurative exudate with WBC 59,200 cells/μL, RBC 28,800 cells/μL (neutrophils 70%, lymphocytes 30%), and Gram‐positive cocci in clusters; culture yielded *Staphylococcus aureus* . The antibiogram showed susceptibility to ciprofloxacin, clindamycin, trimethoprim sulfamethoxazole, doxycycline, and erythromycin. Based on these results, therapy was narrowed to ciprofloxacin 400 mg twice daily and clindamycin 600 mg three times daily, both with documented susceptibility and acceptable intra‐abdominal penetration, together with ulcer therapy.

Focused ultrasound showed no pleural or intraperitoneal free fluid. Ultrasound‐guided percutaneous catheter drainage (PCD) was performed; the catheter subsequently dislodged, and repeat ultrasound‐guided aspiration (without catheter) yielded ~2 mL of purulent material, indicating limited percutaneous control.

In view of persistent biliary inflammatory changes on ultrasound, including a contracted gallbladder with diffuse wall thickening to 7 mm, pericholecystic fat stranding, compact echogenic sludge without cholelithiasis, and a normal proximal common bile duct, the patient first completed a 14‐day *Helicobacter pylori* eradication regimen consisting of metronidazole 500 mg three times daily, clarithromycin 500 mg twice daily, amoxicillin 500 mg twice daily, pantoprazole 40 mg twice daily, and bismuth two tablets daily directed at the duodenal ulcer. He subsequently underwent elective laparoscopic cholecystectomy using a standard four‐port approach. Intraoperatively, the gallbladder was contracted with a uniformly thickened inflamed wall containing sludge and no gross stones, and the wall was intact with no perforation or bile staining. Calot's triangle was edematous, yet the critical view of safety was obtained, the cystic duct and artery were clipped and divided, and the specimen was retrieved in an endoscopic bag. Dense adhesions tethered the inferior surface of hepatic segments IVb and V to the anterior gastric antrum. The antral serosa showed inflammatory change without a visible full‐thickness defect, and no cholecystoenteric fistula, including a cholecystogastric communication, was identified. No drainable hepatic collection was encountered, consistent with ongoing decompression of the liver abscess through the small antral fistulous orifice documented endoscopically; therefore, no abscess drainage was performed. The fistulous origin was not visualized, and given adequate source control, it was managed expectantly to close spontaneously under antibiotics. There was no intraoperative bile leak, the postoperative course was uneventful, and on opening the retrieved gallbladder specimen, microlithiasis was identified. At discharge, the patient received ciprofloxacin 400 mg twice daily and clindamycin 600 mg three times daily.

## 4 Outcome and Follow‐Up

At the first follow‐up visit, the patient was clinically stable and reported adherence to the prescribed medications with gradual improvement in symptoms. On physical examination, the surgical site was well healed with no signs of infection or complications. A comprehensive laboratory panel was obtained, including CBC, ESR, CRP, liver enzymes (AST, ALT, ALP), total and direct bilirubin levels, PT, and INR. All parameters were within normal limits, indicating resolution of the systemic inflammatory response and no evidence of hepatic dysfunction. An abdominal ultrasound focusing on the liver and biliary system was also performed, which demonstrated interval improvement with no signs of recurrent abscess, hepatomegaly, or biliary dilatation. The patient tolerated the treatment well, experienced no adverse drug reactions, and was instructed to continue follow‐up as needed. Further follow‐up evaluations at 1 and 3 months post‐discharge confirmed the patient's continued clinical improvement. At these visits, the patient remained asymptomatic with normal physical examinations and laboratory tests, including complete blood counts and liver function tests, all within normal limits. Overall, the patient achieved complete resolution of symptoms without any residual complaints. No follow‐up cross‐sectional imaging was obtained, given the full clinical recovery and absence of red flags.

## 5 Discussion

Liver abscesses are known to rupture into neighboring anatomical spaces, most commonly the pleural, pericardial, and peritoneal cavities [3]. By contrast, spontaneous fistulization into the gastrointestinal tract, particularly the stomach, is an exceptionally rare complication [5]. A limited number of case reports have documented HGF formation as a sequel to pyogenic or amebic liver abscess. Clinical manifestations in reported cases have varied widely, including melena, bilious vomiting, hematemesis, drainage of ingested food through a percutaneous catheter, or an unexpected reduction in abscess size on follow‐up imaging [6, 7, 8]. Such symptoms are frequently nonspecific or absent, leading to delayed or incidental diagnosis; in our patient, classical gastrointestinal symptoms were lacking, underscoring the diagnostic challenge posed by this entity.

Epidemiologically, PLA is uncommon but its incidence rises sharply with age; population‐based data from North America estimate ~2.3 cases per 100,000 person‐years, with higher risk in men and in individuals with liver transplantation, diabetes, or malignancy [1]. Against this background, our 18‐year‐old patient represents a demographic outlier within typical Western cohorts.

In children and young adults, predisposing conditions differ from those in older adults and may include immunocompromised states (malignancy, malnutrition, chemotherapy/immunosuppression), chronic granulomatous disease, sickle cell disease, diabetes, congenital or acquired biliary tract anomalies, and occasionally abdominal trauma or portal pyemia; pathogen spectra also vary by region. For example, *Staphylococcus aureus* is reported more often in many pediatric series, whereas *Klebsiella pneumoniae* may predominate in parts of Asia [9]. Our patient lacked typical comorbidities, making a biliary‐source PLA in a young host notable. Consistent with this, the sample from the fistulous orifice culture in our case yielded *S. aureus* , and antibiotics were subsequently tailored to the susceptibility profile, a pattern reported more frequently in younger cohorts [10].

Given the limited sensitivity of Charcot's triad, contemporary diagnosis of acute cholangitis follows the Tokyo Guidelines framework. The criteria integrate (A) systemic inflammation, (B) cholestasis, and (C) imaging/etiology; a suspected diagnosis requires A + (B or C), and a definite diagnosis requires A + B + C [11]. In our case, systemic inflammation was present, but neither a cholestatic pattern/jaundice nor biliary dilatation/obstructing lesion was documented at the index imaging. Accordingly, Tokyo criteria for active cholangitis at presentation were not met, and we interpret cholangitis as a probable antecedent event precipitating PLA rather than an ongoing process at the time of evaluation.

**FIGURE 4:**
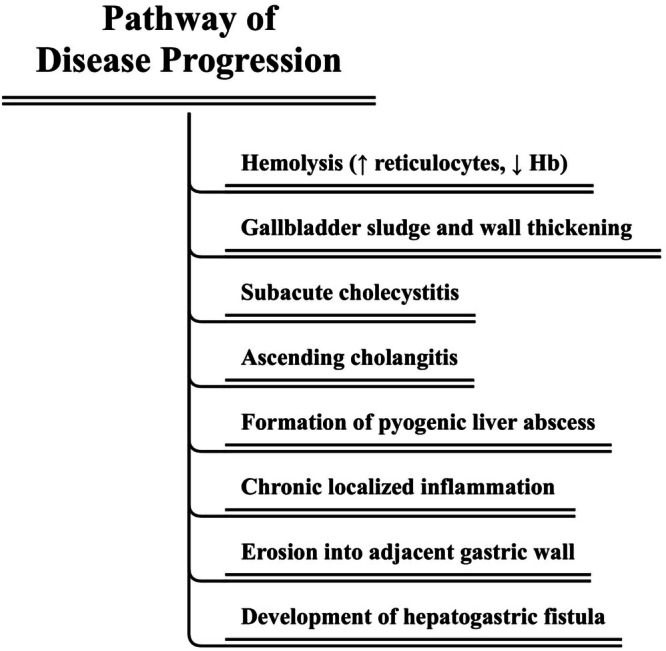
Stepwise pathophysiological progression from hemolysis‐induced biliary sludge to HGF formation.

The pathophysiological cascade in our patient appears to have originated with subclinical hemolysis, as evidenced by an elevated reticulocyte count (6.2%) and low hemoglobin level (9.6 g/dL). Chronic hemolysis is a well‐established risk factor for pigment sludge or stone formation within the biliary system [12], which in this case likely led to biliary sludge accumulation and gallbladder wall thickening, manifesting as subacute cholecystitis. This initial biliary abnormality probably progressed to ascending cholangitis and subsequently to the development of a PLA. Over time, chronic localized inflammation and tissue destruction facilitated fistulous communication between the abscess cavity and the adjacent gastric wall. The occurrence of ascending cholangitis and liver abscess in a previously healthy 18‐year‐old male is itself unusual, given that these conditions more commonly affect older adults [13]. A schematic representation of this hierarchical sequence leading to HGF is provided in Figure 4.

Consistent with this chronology, we interpret the biliary infection/cholangitis as remote (weeks before presentation)—accounting for the absence of ductal dilatation or a cholestatic laboratory pattern at the time of CT. The segment IV–V abscess subsequently eroded into the gastric antrum, explaining the endoscopic antral orifice and the patient's clinical improvement following spontaneous decompression. We also considered primary cholecystogastric perforation with contiguous hepatic involvement. However, there was no CT‐demonstrable cholecystogastric tract or gallbladder perforation, and the topographic concordance between the antral orifice and the segment IV–V collection favors a hepatogastric mechanism. In the absence of confirmatory fistulography and with non‐visualization of the tract intraoperatively, we acknowledge diagnostic uncertainty and present this causal sequence as probable rather than definitive.

The diagnosis of HGF is typically established through a combination of radiological and endoscopic modalities. Contrast‐enhanced CT may demonstrate a hepatic abscess with air–fluid levels and either a visible tract or mural irregularity abutting the gastric antrum [14]. In our case, CT revealed a well‐defined, multiloculated abscess with internal air pockets adjacent to the antrum, without a discrete tract, raising suspicion for fistulization; subsequent EGD confirmed a small antral orifice with purulent drainage, thereby establishing gastric involvement.

Timely and adequate drainage remains central to PLA management to mitigate complications such as rupture or fistulization. Prior reports emphasize that early intervention reduces adverse outcomes [15, 16]. While no standardized treatment guidelines currently exist for HGF [4], the management approach generally depends on the patient's clinical stability, extent of the fistula, and presence of complications. Surgical repair remains the definitive treatment in many cases, especially when conservative management fails or complications such as hemorrhage or persistent sepsis occur [17]. Image‐guided drainage is the first‐line intervention for PLA and generally outperforms single needle aspiration in treatment success; however, surgery is appropriate when percutaneous drainage is infeasible, inadequate, or complicated by concurrent intra‐abdominal pathology requiring operative source control. In our patient, the abscess was large and multiloculated, and early catheter dislodgement yielded minimal evacuation (~2 mL), indicating limited percutaneous control. Concomitant gallbladder pathology also favored definitive source control via cholecystectomy with abscess drainage, consistent with published management strategies [18]. Nevertheless, there are reports of successful conservative management using broad‐spectrum antibiotics and proton pump inhibitors, with spontaneous closure of the fistula [8]. In cases presenting with upper gastrointestinal bleeding, angioembolization has been proposed as a hemostatic measure [19].

## 6 Limitations

Lack of confirmatory fistulography/cholecystography and absence of follow‐up imaging are acknowledged limitations; nevertheless, the concordant clinical course and laboratory normalization after source control are consistent with resolution.

## 7 Conclusion

This report presents HGF as a rare complication of PLA in a young patient. It underscores the diagnostic challenges of atypical presentations and highlights the complementary roles of cross‐sectional imaging and endoscopy. Early recognition and a multidisciplinary approach combining antimicrobial therapy, drainage, and, when indicated, elective surgical source control are essential for favorable outcomes. Documenting such uncommon complications enhances clinical awareness and may inform future management.

## Author Contributions

**Mohammad Kazem Amirbeigy:** conceptualization, formal analysis, investigation, methodology, supervision, writing – review and editing. **Amir Pasha Amel Shahbaz:** formal analysis, investigation, methodology, writing – review and editing. **Zahra Sekandari:** writing – original draft. **Shahab Sheikhalishahi:** data curation, visualization, writing – original draft, writing – review and editing.

## Ethics Statement

The authors are accountable for all aspects of the work in ensuring that questions related to the accuracy or integrity of any part of the work are appropriately investigated and resolved. All procedures involving human participants were in accordance with the ethical standards of the institutional and/or national research committees and with the Helsinki Declaration (2013 revision) [20]. Written informed consent was obtained from the patient for the publication of the case report and any accompanying images.

## Conflicts of Interest

The authors declare no conflicts of interest.

## Data Availability

The clinical data related to this case report are available on request from the corresponding author. The data are not publicly available due to privacy and ethical restrictions.
